# Supplementary material for: Robust upward dispersion of the neutron spin resonance in the heavy fermion superconductor Ce1−xYbxCoIn5
Source: Nat Commun. 2016 Sep 28;7:12774. doi: 10.1038/ncomms12774 (PMC5052703; doi:10.1038/ncomms12774)
Supplement: Supplementary Information — Supplementary Figures 1-15, Supplementary Notes 1-5 and Supplementary References [file ncomms12774-s1.pdf]

Supplementary Information:

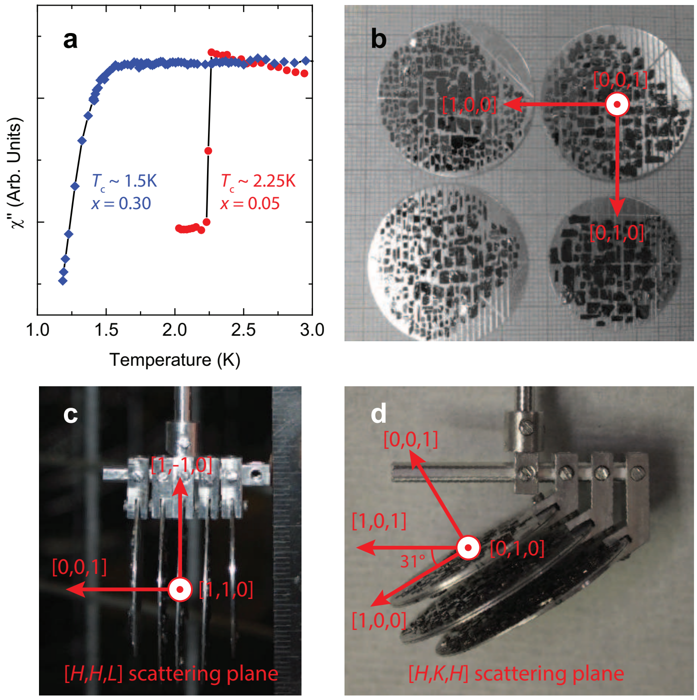

Supplementary Figure 1: Measurement of T_c and co-alignment of $Ce_{1-x}Yb_xCoIn_5$ single crystals. (a) AC magnetic susceptibility measured on $Ce_{1-x}Yb_xCoIn_5$ ($x = 0.05$ and 0.3), with T_c =2.25 K and 1.5 K. (b) Several aluminum plates with hundreds of co-aligned $Ce_{1-x}Yb_xCoIn_5$ ($x = 0.05$) single crystals. The crystallographic axes are marked by red arrows. (c) Co-aligned plates in the $[H, H, L]$ scattering plane. (d) Co-aligned plates in the $[H, K, H]$ scattering plane. The angle between $[1, 0, 1]$ and $[1, 0, 0]$ is $\sim 31^{\circ}$.

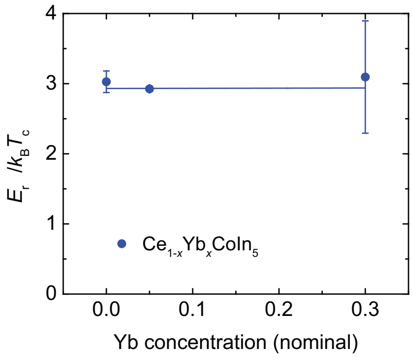

Supplementary Figure 2: E_r/k_BT_c in $Ce_{1-x}Yb_xCoIn_5$. The result for $CeCoIn_5$ is obtained from previous work [1] and the results for $x = 0.05$ and $x = 0.3$ are from this work. The vertical error bars are estimates of the uncertainty of E_r by carrying out constant-$\mathbf{Q}$ scans at $\mathbf{Q}_{AF} = (0.5, 0.5, 0.5)$.

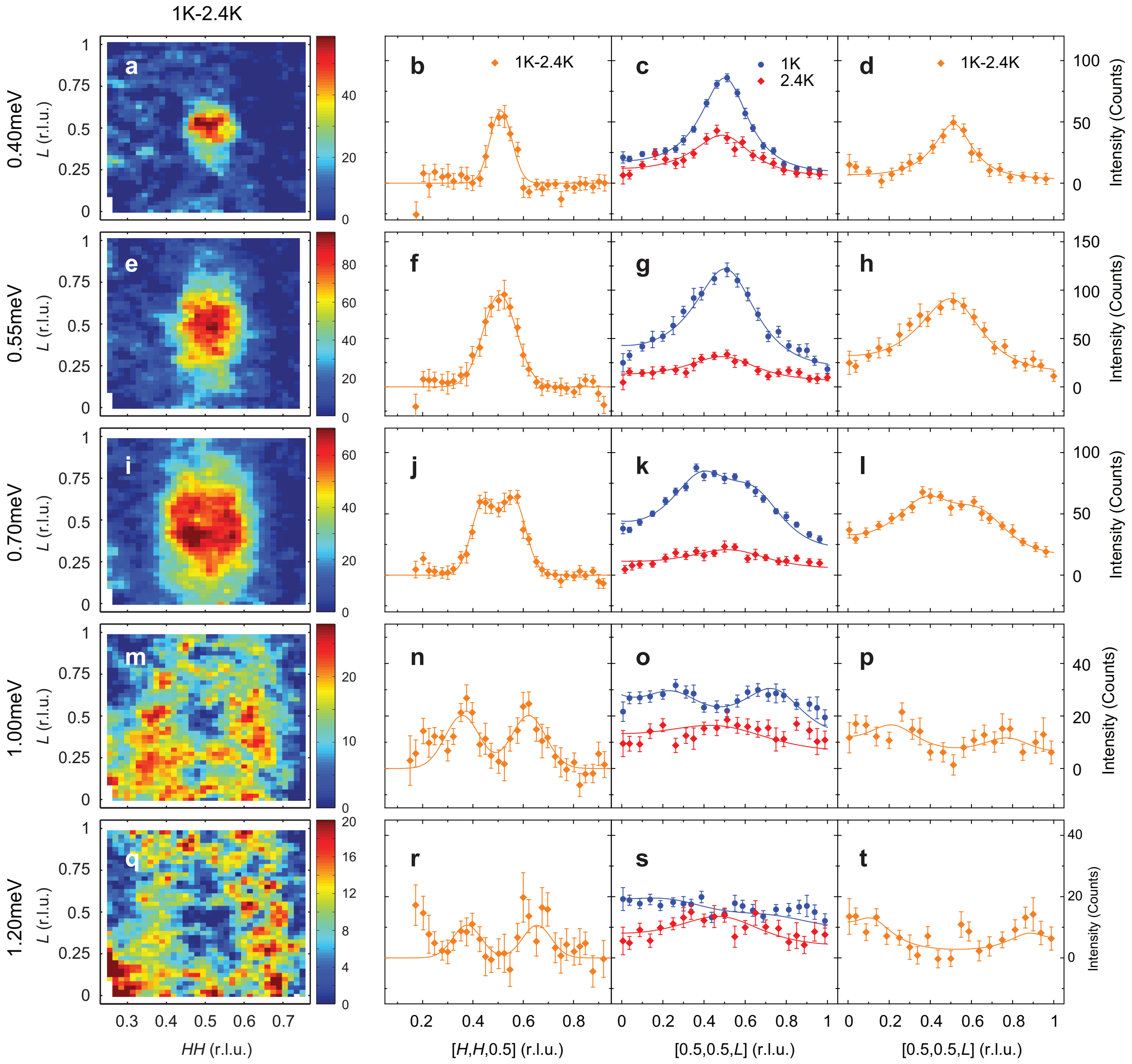

Supplementary Figure 3: Additional neutron scattering data for $Ce_{0.95}Yb_{0.05}CoIn_5$. (a) Constant-energy map for $Ce_{0.95}Yb_{0.05}CoIn_5$ at $E = 0.4$ meV and 1 K after subtracting data from 2.4 K. (b) Cut along $[H, H, 0.5]$ for the map in (a) at $E = 0.4$ meV, the solid line is a fit assuming zero background. (c) Cuts along $[0.5, 0.5, L]$ at both 1 K and 2.4 K obtained from maps in Figure 3(a) and (b) in the main text. (d) Cut along $[0.5, 0.5, L]$ for the map in (a) at 1K after subtracting data from 2.4 K. The solid lines in (c) and (d) are sums of Lorentzian peaks polarized along $\hat{\mathbf{c}}$. Similarly (e), (f), (g) and (h) are for $E = 0.55$ meV, (i), (j), (k) and (l) are for $E = 0.7$ meV, (m), (n), (o) and (p) are for $E = 1.00$ meV and (q), (r), (s) and (t) are for $E = 1.20$ meV. For cuts along $[0.5, 0.5, L]$ at 2.3 K and $E = 0.40$ and 0.55 meV, $F(L)$ is a single Lorentzian peak centered at $L = 0.5$. For 1 K and 1 K-2.4 K cuts with $E = 0.7, 1.0$ and 1.20 meV, $F(L)$ is two Lorentzian peaks equally displaced from $L = 0.5$. All vertical error bars in the Figure represent statistical errors of 1 standard deviation.

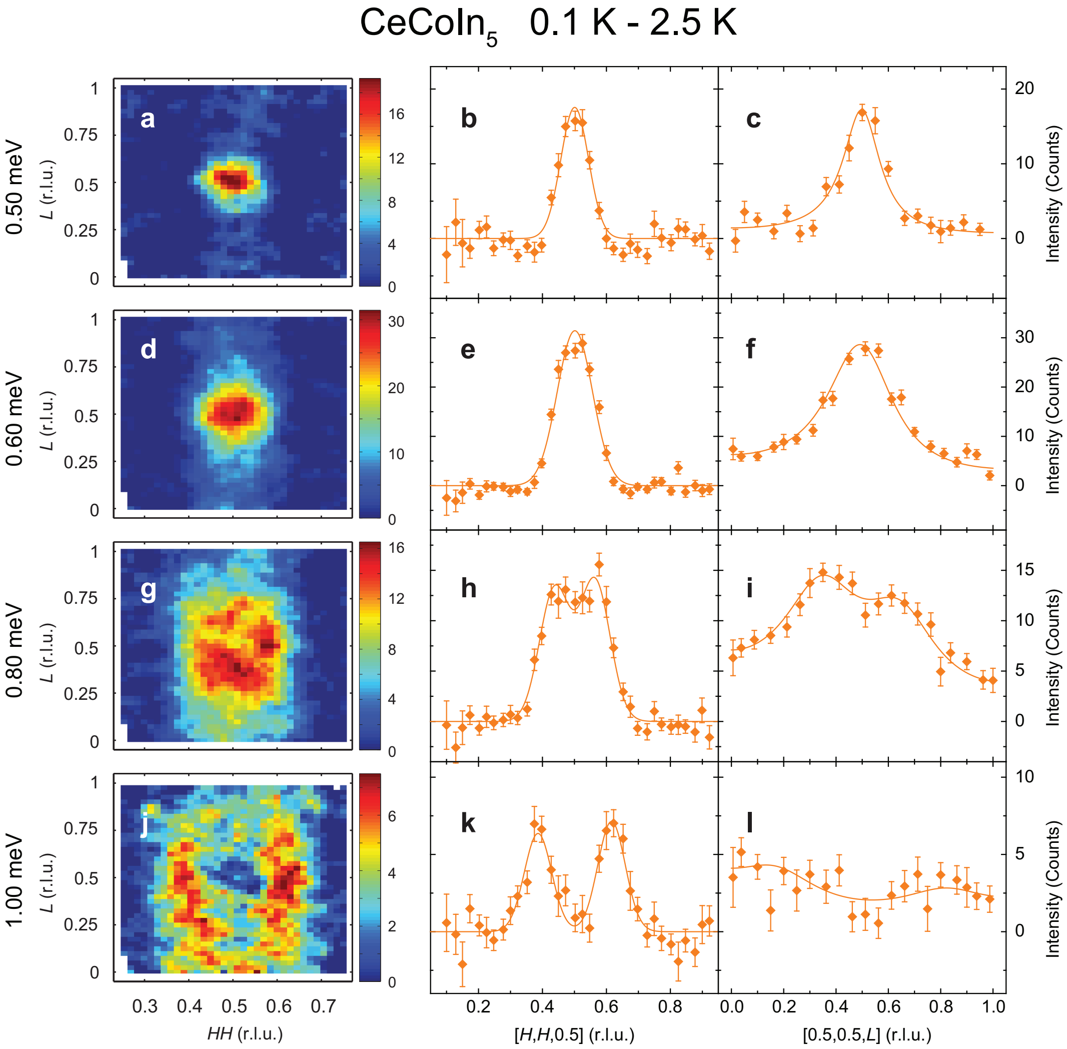

Supplementary Figure 4: Neutron scattering data for $CeCoIn_5$. (a) Constant-energy map for $CeCoIn_5$ at $E = 0.5$ meV and 0.1 K after subtracting data from 2.5 K. (b) Cut along $[H, H, 0.5]$ for the map in (a), the solid line is a fit assuming zero background. (c) Cut along $[0.5, 0.5, L]$ for the map in (a), the solid line is a sum of Lorentzian peaks polarized along $\hat{\mathbf{c}}$. Similarly (d), (e) and (f)are for $E = 0.6$ meV, (g), (h) and (i) are for $E = 0.8$ meV and (j), (k) and (l) are for $E = 1.00$ meV. For cuts along $[0.5, 0.5, L]$ for $E = 0.50$ and 0.60 meV, $F(L)$ is a single Lorentzian peak centered at $L = 0.5$. For $E = 0.8$ and 1.0 meV, $F(L)$ is two Lorentzian peaks equally displaced from $L = 0.5$. All vertical error bars in the Figure represent statistical errors of 1 standard deviation.

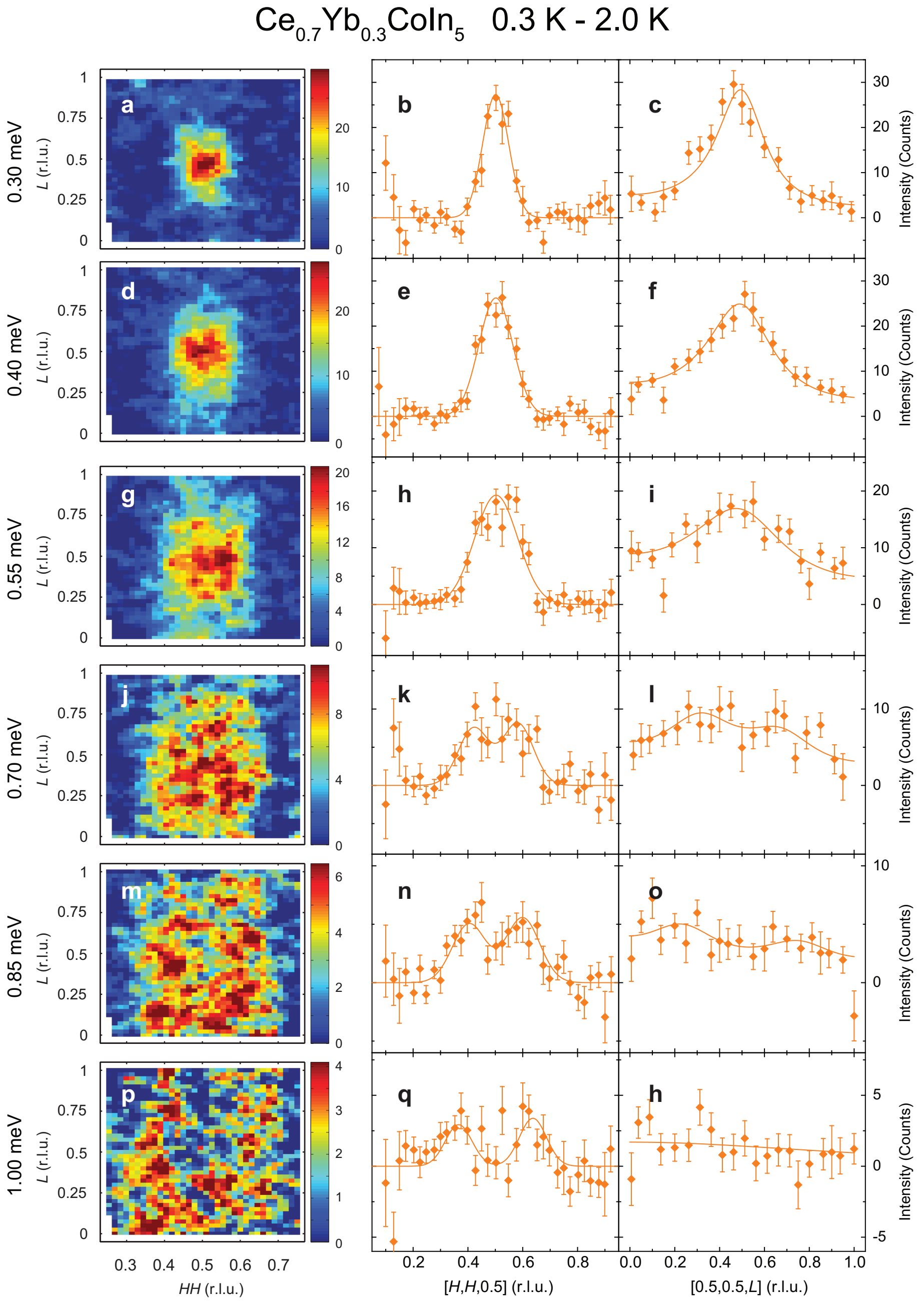

Supplementary Figure 5: Neutron scattering data for $Ce_{0.7}Yb_{0.3}CoIn_5$. (a) Constant-energy map for $Ce_{0.7}Yb_{0.3}CoIn_5$ at $E = 0.3$ meV and 0.3 K after subtracting data from 2.0 K. (b) Cut along $[H, H, 0.5]$ for the map in (a), the solid line is a fit assuming zero background. (c) Cut along $[0.5, 0.5, L]$ for the map in (a), the solid line is a sum of Lorentzian peaks polarized along $\hat{\mathbf{c}}$. Similarly (d), (e) and (f)are for $E = 0.4$ meV, (g), (h) and (i) are for $E = 0.55$ meV, (j), (k) and (l) are for $E = 0.7$ meV, (m), (n) and (o) are for $E = 0.85$ meV and (p), (q) and (r) are for $E = 1.0$ meV. For cuts along $[0.5, 0.5, L]$ for $E = 0.3$, 0.4 and 0.55 meV, $F(L)$ is a single Lorentzian peak centered at $L = 0.5$. For $E = 0.7$, 0.85 and 1.0 meV, $F(L)$ is two Lorentzian peaks equally displaced from $L = 0.5$. All vertical error bars in the Figure represent statistical errors of 1 standard deviation.

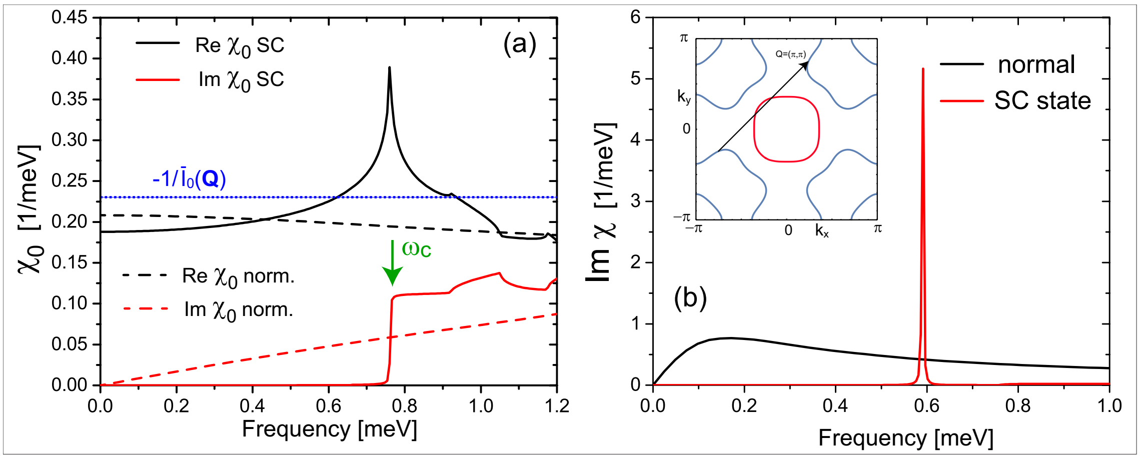

Supplementary Figure 6: χ in the spin-exciton scenario at $\mathbf{Q}_{\mathrm{AF}}$. (a) Real and imaginary parts of χ_0 at $\mathbf{Q}_{\mathrm{AF}}$ in the normal (dashed lines) and superconducting (solid lines) state of CeCoIn_5. The onset energy ω_c of the particle-hole continuum in the superconducting state is shown by a green arrow. The blue dotted line indicates the value of $-1/\bar{I}_0(\mathbf{Q})$, such that its intersection with Re χ_0 in the superconducting state for $\omega < \omega_c$ yields the position of the spin exciton. (b) Full χ in the normal and superconducting state at $\mathbf{Q}_{\mathrm{AF}}$. Inset: Fermi surface and scattering vector $\mathbf{Q}_{\mathrm{AF}}$ between momentum states on the Fermi surface.

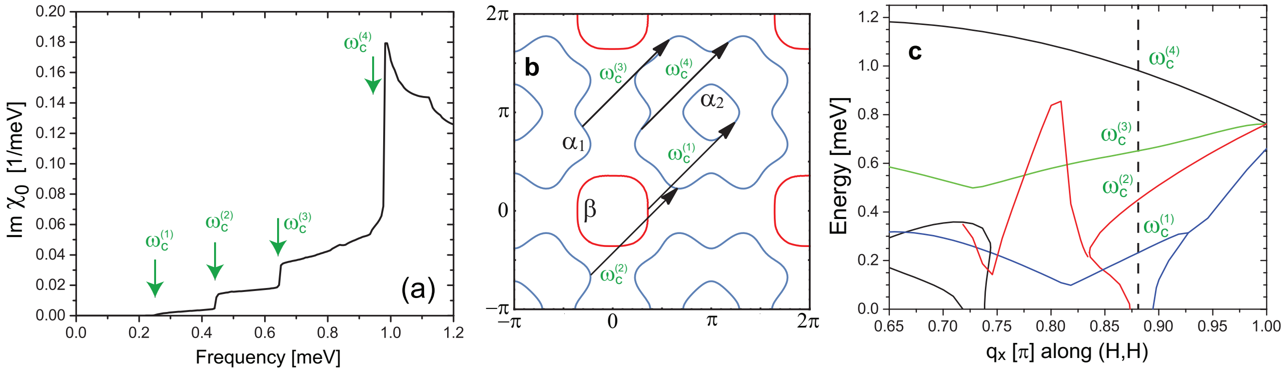

Supplementary Figure 7: χ in the spin-exciton scenario at $0.88\mathbf{Q}_{\mathrm{AF}}$. (a) Imaginary parts of χ_0 at $\mathbf{q} = 0.88\mathbf{Q}_{\mathrm{AF}}$ in the superconducting state of CeCoIn_5. The onset energies $\omega_c^{(i)}$ of the particle-hole continuum in the superconducting state are shown by green arrows. (b) Fermi surfaces in the extended Brillouin zone with scattering vectors corresponding to $\omega_c^{(i)}$ in (a). (c) Momentum dependence of the onset energies $\omega_c^{(i)}$ along the [1,1,0] direction. The dashed line corresponds to the momentum $\mathbf{q} = 0.88\mathbf{Q}_{\mathrm{AF}}$ for which Im χ_0 is shown in (a), with the indicated $\omega_c^{(i)}$ being the same as in (a).

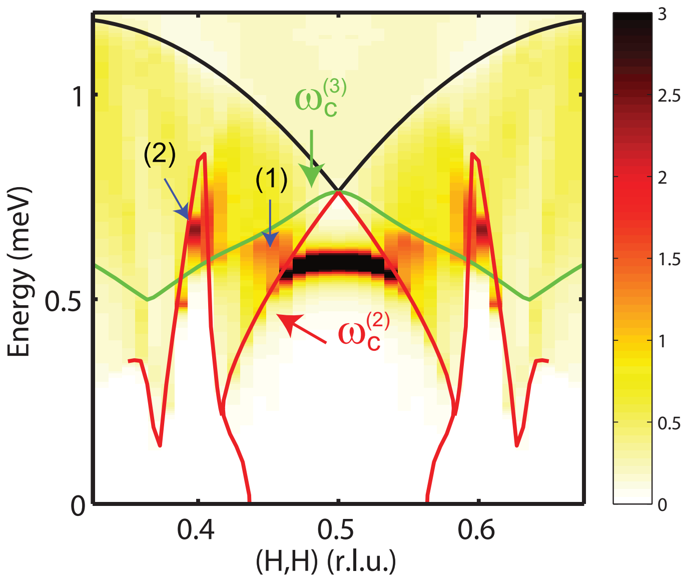

Supplementary Figure 8: Momentum dependence of the resonance as a spin-exciton with some of the onset energies of Fig. 7(c) overlain as solid lines.

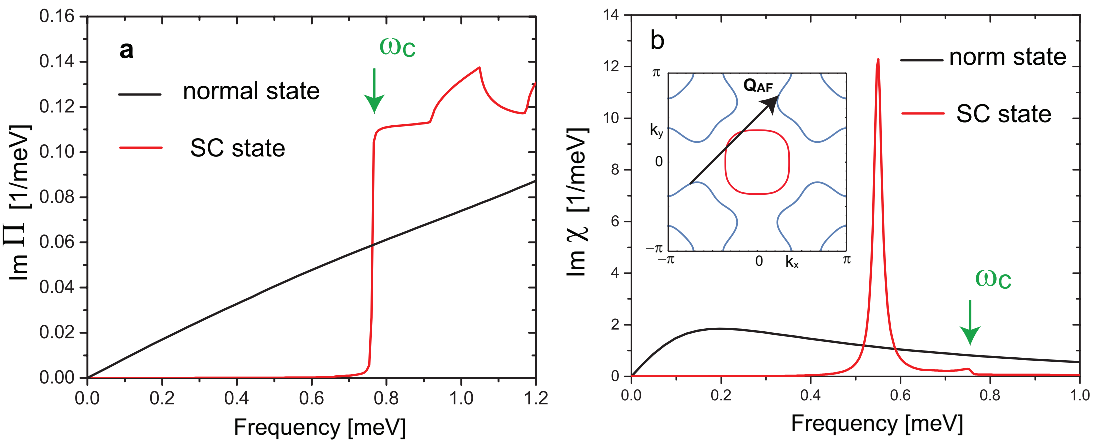

Supplementary Figure 9: The resonance as a magnon-like excitation at $\mathbf{Q}_{\mathrm{AF}}$. (a) Imaginary parts of Π at $\mathbf{Q}_{\mathrm{AF}}$ in the normal (black line) and superconducting (red line) state of CeCoIn_5. The onset energy ω_c of the particle-hole continuum in the superconducting state is shown by a green arrow. (b) Full χ in the normal (black line) and superconducting (red line) state $\mathbf{Q}_{\mathrm{AF}}$. The resonance occurs at $\omega = \Delta_{\mathrm{sw}}$ below ω_c (see green arrow). Inset: Scattering process contributing to Im Π.

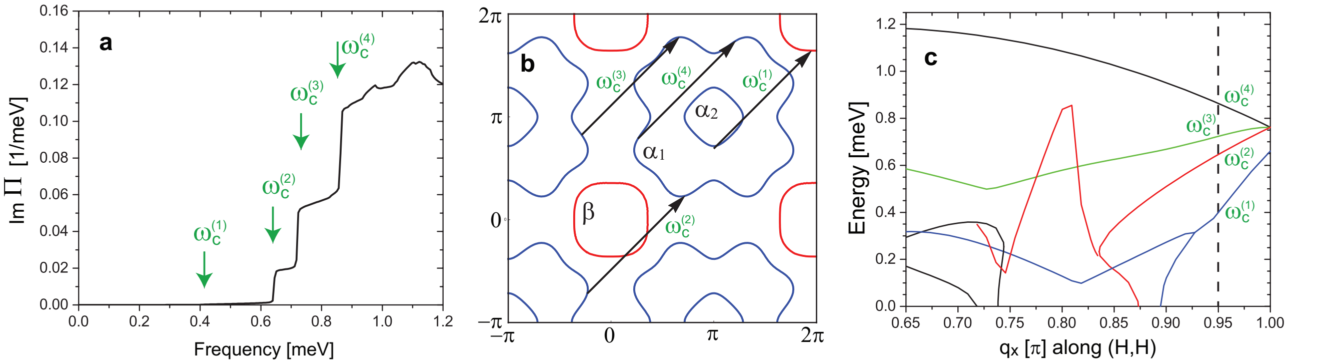

Supplementary Figure 10: The resonance as a magnon-like excitation at $0.95\mathbf{Q}_{\mathrm{AF}}$. (a) Imaginary part of Π in the superconducting state of $CeCoIn_5$ at $\mathbf{q} = 0.95\mathbf{Q}_{\mathrm{AF}}$. The onset energies $\omega_c^{(i)}$ for particle-hole scattering in the superconducting state are shown by green arrows. (b) Fermi surfaces in the extended Brillouin zone with scattering vectors corresponding to $\omega_c^{(i)}$ in (a). (c) Momentum dependence of $\omega_c^{(i)}$ along $q_x = q_y$. The dashed line corresponds to the momentum $\mathbf{q} = 0.95\mathbf{Q}_{\mathrm{AF}}$ for which Im Π is shown in (a), with the indicated $\omega_c^{(i)}$ being the same as in (a).

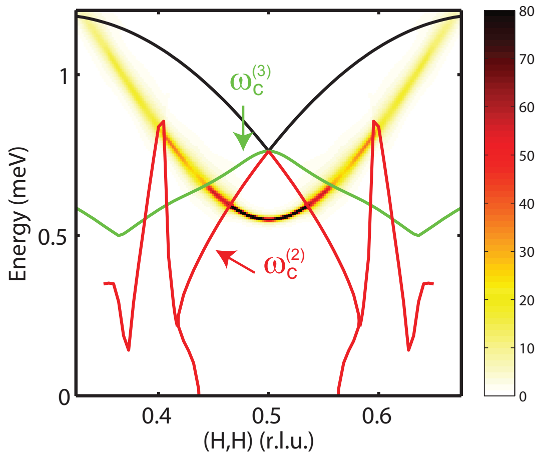

Supplementary Figure 11: Momentum dependence of the resonance as a magnon-like excitation with some the onset energies of SFig. 10(b) overlain as solid lines.

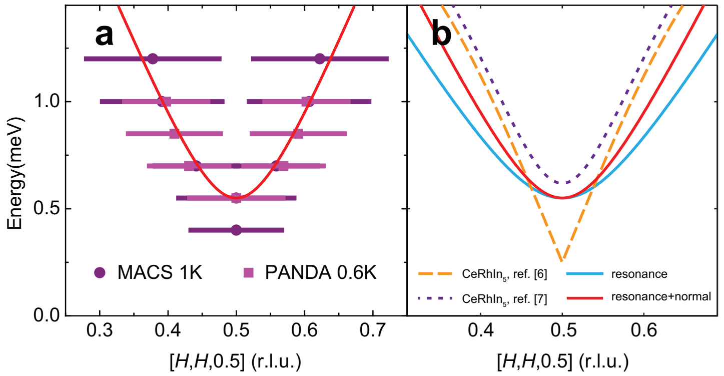

Supplementary Figure 12: Comparison of dispersive magnetic excitations in $Ce_{0.95}Yb_{0.05}CoIn_5$ with spin waves in $CeRhIn_5$. (a) Dispersion of magnetic excitations along $[H, H, 0.5]$ in $Ce_{0.95}Yb_{0.05}CoIn_5$ in the superconducting state obtained at 0.6 K on PANDA and 1 K on MACS. The points are obtained from scans in Figure 2(c) and Figure 3(c), (f), (i), (l) and (o). The solid red curve is the fit to $E = \sqrt{\Delta^2 + (c\mathbf{q})^2}$ with $\Delta = 0.55(1)$ meV and $c = 4.0(1)$ meV$\cdot$ Å. (b) Comparison of dispersion of magnetic excitations in $CeRhIn_5$ and $Ce_{0.95}Yb_{0.05}CoIn_5$, similar to Figure 1(j) but with the red curve from (a) also added in for comparison. The response in the superconducting state can be thought of as the sum of the resonance mode and normal state excitations and since the normal state response are broad peaks centered at $\mathbf{Q}_{\rm AF}$, the dispersion of the magnetic excitations in the superconducting state (resonance plus normal state excitations) has a larger velocity than the resonance itself.

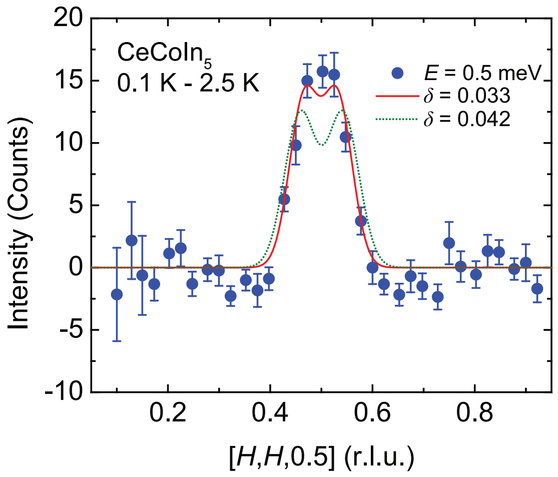

Supplementary Figure 13: Peak width of the resonance in $CeCoIn_5$ at $E = 0.5$ meV. Constant-energy scan along $[H, H, 0.5]$ centered at $\mathbf{Q}_{\rm AF}$ for $E = 0.50$ meV, obtained by subtracting 2.5 K data from 0.1 K data for $CeCoIn_5$. The cut is obtained by binning data with $0.42 < L < 0.58$. The data is fit with two Gaussian peaks separated by 2δ. The dashed green line shows the best fit by fixing $\delta = 0.042$, and the solid red line is the best fit by fixing $\delta = 0.033$.

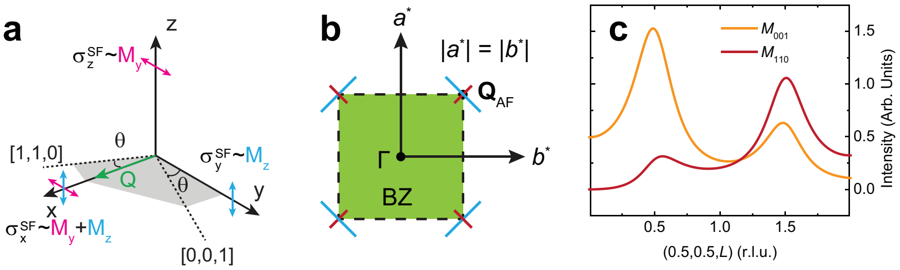

Supplementary Figure 14: Possible contribution to the resonance polarized along $[1, 1, 0]$. (a) Scattering geometry of polarized neutron scattering experiment used in previous work [4]. (b) Schematic of the reciprocal space for $CeCoIn_5$ with tetragonal symmetry. The green box indicate the first Brillouin zone, unlike Γ, anisotropy at $\mathbf{Q}_{\mathrm{AF}}$ = (0.5,0.5) between the longitudinal direction ($[1, 1, 0]$, red solid line) and the tranverse direction ($[1, \bar{1}, 0]$, blue solid line) does not break the four-fold rotational symmetry of the underlying lattice. (c) Comparison of M_{110} and M_{001} at $\mathbf{Q}_{\mathrm{AF}}$ = (0.5,0.5,0.5). In unpolarized neutron scattering measurements, M_{110} contributes little to the overall intensity near L = 0.5 but becomes significant near L = 1.5, where absorption becomes strong.

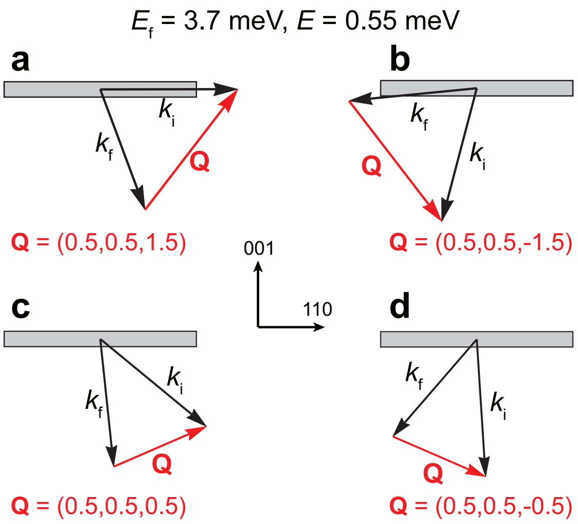

Supplementary Figure 15: Scattering geometries at several different wave vectors. (a) Scattering triangle for $\mathbf{Q}$ = (0.5,0.5,1.5) for $E_{\mathrm{f}} = 3.7$ meV and $E = 0.55$ meV, the slab represents the sample array which is much longer along $[1, 1, 0]$ than $[0, 0, 1]$. (b), (c) and (d) show similar scattering triangles for $\mathbf{Q}$ = (0.5,0.5,-1.5), (0.5,0.5,0.5) and (0.5,0.5,-0.5).

Supplementary Note 1: The Magnetic Resonance as a Spin Exciton

Within the spin-exciton scenario [5–14], a magnetic resonance emerges below T_c as a feedback effect of the unconventional superconducting order parameter on the spin excitation spectrum. This feedback effect can be easily understood by considering the full spin susceptibility in the random-phase approximation (RPA) which is given by [see Supplementary Eq. 42 in Ref.[15]]

$$\chi(\mathbf{q}, \omega) = \frac{1}{2} \frac{\chi_0(\mathbf{q}, \omega)}{1 + \bar{I}_0(\mathbf{q})\chi_0(\mathbf{q}, \omega)} \tag{S1}$$

where χ_0 is the non-interacting susceptibility and $\bar{I}_0(q)$ is the bare magnetic interaction, which was extracted in Ref. [15]. We employ Supplementary Eq. 39 in Ref.[15] to compute $\chi_0(\mathbf{q}, \omega)$. To understand the emergence of a resonance, we begin by considering the form of χ_0 at $\mathbf{q} = \mathbf{Q}_{\mathrm{AF}}$ in the normal and superconducting states, as shown in Supplementary Fig. 6(a).

In the normal state, $\mathrm{Im}\chi_0$ increases linearly, while $\mathrm{Re}\chi_0$ is featureless and decreases monotonically. In contrast, in the superconducting state, $\mathrm{Im}\chi_0$ vanishes below an onset energy $\omega_c(\mathbf{Q}_{\mathrm{AF}})$ where it exhibits a sharp jump. Similar to

the discussion in the magnon-like scenario below, the scattering of electrons from $\mathbf{k}$ to $\mathbf{k}+\mathbf{Q}_{\rm AF}$ requires the breaking of Cooper pairs and thus a minimum energy – the onset energy for non-zero Imχ_0 – given by $\omega_c(\mathbf{Q}_{\rm AF}) = |\Delta_{\mathbf{k}}|+|\Delta_{\mathbf{k}+\mathbf{Q}_{\rm AF}}|$. The corresponding scattering process is shown in the inset of Supplementary Fig. 6(b). As previously pointed out, the sharp jump of Imχ_0 at $\omega_{\rm c}$ arises since the scattering process involves momentum states on the Fermi surface that possess opposite signs of the superconducting gap. As a result, Reχ_0 exhibits a logarithmic divergence at $\omega_{\rm c}$ and the resonance condition $1 + \bar{I}_0(\mathbf{Q}_{\rm AF})\mathrm{Re}\chi_0(\mathbf{Q}_{\rm AF}, \omega_{\rm R}) = 0$ can be satisfied at a frequency $\omega_{\rm R} < \omega_{\rm c}$ for arbitrary small interaction $\bar{I}_0(\mathbf{Q}_{\rm AF})$, as follows from the intersection of $-1/\bar{I}_0(\mathbf{Q}_{\rm AF})$ and Reχ_0 shown in Supplementary Fig. 6(a). At the same time, $\mathrm{Im}\chi_0(\mathbf{Q}_{\rm AF}, \omega_{\rm R})$ vanishes as well (since $\omega_{\rm R} < \omega_{\rm c}$), giving rise to a sharp peak in $\mathrm{Im}\chi(\mathbf{Q}_{\rm AF}, \omega)$ at $\omega_{\rm R}$.

For momenta away from $\mathbf{Q}_{\rm AF}$, there exist multiple scattering channels for the same scattering vector $\mathbf{q}$ with different onset energies, $\omega_{\rm c}^{(i)}(\mathbf{q})$, as follows from a plot of Imχ_0 as a function of frequency at $\mathbf{q} = 0.88\mathbf{Q}_{\rm AF}$ in Supplementary Fig. 7(a). The scattering vectors corresponding to the various onsets are shown in Supplementary Fig. 7(b), while the momentum dependence of $\omega_{\rm c}^{(i)}(\mathbf{q})$ along the diagonal direction is shown in Supplementary Fig. 7(c).

According to the above discussion, no resonance can exist below $\omega_{\rm c}^{(1)}$, due to the absence of a logarithmic divergence associated with $\omega_{\rm c}^{(1)}$. This is confirmed by a plot of Imχ together with the onset energies along the diagonal direction, shown in Supplementary Fig. 8. Moreover, as expected we find that in the vicinity of $\mathbf{Q}_{\rm AF}$, a resonance exists only below $\omega_{\rm c}^{(2)}$, due to the large value of Imχ_0 for $\omega > \omega_{\rm c}^{(2)}$. However, away from $\mathbf{Q}_{\rm AF}$, weaker resonances can exist even above $\omega_{\rm c}^{(2)}$ [see arrow (1)] or $\omega_{\rm c}^{(3)}$ [see arrow (2)] due to a smaller Imχ_0 at higher energies.

Supplementary Note 2: The Magnetic Resonance as an Undamped Paramagnon

The close proximity of superconductivity and the AF state in the cuprate superconductors had previously given rise to the suggestion that the magnetic resonance is a paramagnon that becomes undamped below T_c due to the opening of the particle-hole continuum. The suggestion that $CeCoIn_5$ is close to an AF instability [16] raises the possibility that a similar scenario might be realized here. To explore this idea, we assume that the paramagnon dispersion is given by

$$\omega_{\rm sw}^2(\mathbf{q}) = \Delta_{\rm sw}^2 + c_{\rm sw}^2(\mathbf{q} - \mathbf{Q}_{\rm AF})^2 \tag{S2}$$

where $\Delta_{\rm sw}$ is the spin-wave gap and $c_{\rm sw}$ is the spin-wave velocity. In the paramagnetic state, $\Delta_{\rm sw} = c_{\rm sw}/\xi$ where ξ is the magnetic correlation length. The presence of a magnetic anisotropy modifies $\Delta_{\rm sw}$ and can lead to different values of $\Delta_{\rm sw}$ for different spin polarizations. Above T_c, the paramagnon is strongly damped due to its coupling to particle-hole excitations. However, in the superconducting state, the mode can become undamped if $\Delta_{\rm sw}$ is smaller than the onset energy for the particle hole continuum. The values of $\Delta_{\rm sw}$ can be T_c and sample dependent. For example, La-doping to $CeCoIn_5$ suppresses T_c and expands the lattice [17]. This process may bring $Ce_{1-x}La_xCoIn_5$ closer to AF ordered phase with a reduced $\Delta_{\rm sw}$ and T_c. Similarly, Yb-doped $CeCoIn_5$ may also be closer to AF ordered phase with reduced T_c and $\Delta_{\rm sw}$.

To further explore this idea, we employ the spin-fermion scenario [18] where the spin-propagator is given by

$$\chi^{-1} = \bar{\chi}^{-1} - \Pi \tag{S3}$$

with $\bar{\chi}$ being the bare spin propagator and Π is the irreducible polarization operator. Since Re $\chi^{-1} = \bar{\chi}^{-1} - \mathrm{Re}\Pi$ is determined by electronic excitations at all energies, it cannot be computed within the current model. We therefore will use the phenomenological form

$$\mathrm{Re}\chi^{-1} = \bar{\chi}^{-1} - \mathrm{Re}\Pi = \frac{\omega_{\rm sw}^2(\mathbf{q}) - \omega^2}{\alpha} \tag{S4}$$

with the parameters in $\omega_{\rm sw}^2(\mathbf{q})$ being determined to correctly reproduce the dispersion of the experimentally observed resonance mode with $\Delta_{\rm sw} = 0.5498$ meV and $c_{\rm sw} = 3.2463$ Å^{-1}. Here, α reflects the (in general momentum dependent) spectral weight of the mode above T_c. We assume that the opening of the superconducting gap below T_c will not change the above form. Within this scenario, the main effect on the spin excitation spectrum in the superconducting state arises from ImΠ, which describes the damping of the spin excitations due to their decay into particle-hole excitations. To lowest order in the spin-fermion coupling g, the polarization operator is given by

$$\Pi = g^2\chi_0 \tag{S5}$$

where χ_0 is the bare susceptibility introduced in Supplementary Equation 39 of Ref. [15] (for the calculation of χ_0, we take the same parameters as in Ref. [15]), and g is the strength of the electronic coupling to the spin-wave mode. Below, we use for concreteness $g^2 = 20.0$ meV^2, however, we note that the specific value of g^2 does not affect the position of the resonance, only its width. In Supplementary Fig. 9(a) we plot ImΠ at $\mathbf{Q}_{\rm AF}$ both in the normal and superconducting state. While ImΠ increases linearly in the normal state, it vanishes below a certain onset energy, $\omega_{\rm c}$, in the superconducting state. This onset energy arises since the decay of the spin excitation into a particle-hole pair with momenta $\mathbf{k}$ and $\mathbf{k} + \mathbf{Q}_{\rm AF}$ (which both lie on the Fermi surface) requires a minimum energy given by $\omega_{\rm c}(\mathbf{Q}_{\rm AF}) = |\Delta_{\mathbf{k}}| + |\Delta_{\mathbf{k}+\mathbf{Q}_{\rm AF}}|$. The corresponding scattering process is shown in the inset of Supplementary Fig. 9(b). If $\Delta_{\rm sw} < \omega_{\rm c}(\mathbf{Q}_{\rm AF})$, the spin mode becomes undamped in the superconducting state, and Im χ exhibits a significant increase in intensity, as shown in Supplementary Fig. 9(b).

Away from $\mathbf{Q}_{\rm AF}$, several scattering channels with different onset energies emerge, as shown in Supplementary Fig. 10(a) where we plot ImΠ as a function of energy at $\mathbf{q} = 0.95\mathbf{Q}_{\rm AF}$. The lowest energy onset, $\omega_{\rm c}^{(1)}$, arises from a scattering channel that connects the α_2 and β Fermi surfaces as shown in Supplementary Fig. 10(b). Since this scattering channel connects momentum points $\mathbf{k}$ and $\mathbf{k} + \mathbf{q}$ on the Fermi surfaces that possess the same sign of the superconducting gap, ImΠ increases linearly in energy above $\omega_{\rm c}^{(1)}$, and does not exhibit a sharp jump. The three remaining higher energy onsets all connect momenta on the $\alpha_{1,2}$ Fermi surfaces, with opposite phase of the superconducting order parameter, hence leading to sharp jumps in Imχ_0 at the corresponding $\omega_{\rm c}^{(2-4)}$. The momentum dependence of these onset energies along the diagonal direction is shown in Supplementary Figs. 10(c). While the energy of the paramagnon lies always above $\omega_{\rm c}^{(1)}$, ImΠ is rather small for energies $\omega_{\rm c}^{(1)} < \omega < \omega_{\rm c}^{(2)}$, such that the paramagnon is only very weakly damped for energies $\omega < \omega_{\rm c}^{(2)}$, but becomes increasingly damped as its energy crosses $\omega_{\rm c}^{(2,3)}$.

To demonstrate this, we present in Supplementary Fig. 11 an intensity plot of Imχ as a function of frequency and momentum along the diagonal direction together with the onset energies, $\omega_{\rm c}^{(2,3)}$. Clearly, the intensity of the resonance mode decreases as its energy crosses $\omega_{\rm c}^{(2,3)}$.

Supplementary Note 3: Dispersion of the magnetic excitations in the superconducting state

In Figure 1(d)-(f), dispersion of the resonance mode is plotted for $Ce_{0.95}Yb_{0.05}CoIn_5$ (as determined from the difference of the data well below and above T_c). In Supplementary Figure 12(a), the dispersion of the magnetic excitations in the superconducting state is plotted (as determined from the data well below T_c). Since the normal state magnetic excitations are broad peaks centered at $\mathbf{Q}_{\rm AF}$, the dispersion obtained after subtracting the normal state response disperses slower compared to the dispersion obtained from the superconducting state alone (sum of the resonance mode and the normal state response). By fitting the dispersion in Supplementary Figure 12(a) with $E = \sqrt{\Delta^2 + (c|\mathbf{q}|)^2}$ (solid red line), we find $\Delta = 0.55(1)$ meV and $c = 4.0(1)$ meV·Å compared to $\Delta = 0.55(1)$ meV and $c = 3.2(1)$ meV·Å in Figure 1(d)-(f). This value of c is closer to $c = 4.8(2)$ meV·Å [2] and $c \approx 5.2$ meV·Å [3] in $CeRhIn_5$.

The magnon-like resonance mode in $Ce_{0.95}Yb_{0.05}CoIn_5$ therefore is slightly softened compared to spin waves in $CeRhIn_5$. This behavior is very different from the upward dispersing resonance found in iron pnictides, where the dispersion of the resonance [$c_{\rm res} = 50(5) - 85(5)$meV·Å] is much softer than the spin waves ($c \approx 450$ meV·Å) [19], this is not surprising considering the upward dispersing resonance in iron pnictides can be interpreted as a weak coupling spin-exciton. The comparison of dispersion for spin waves in $CeRhIn_5$ and magnetic excitations in $Ce_{0.95}Yb_{0.05}CoIn_5$ is shown Supplementary Figure 12(b), which is similar to Figure 1(j) but has the solid red line for the dispersion of the magnetic excitations in the superconducting state (resonance+normal) shown as well.

Supplementary Note 4: Incommensurate excitations at $E = 0.5$ meV

Recently, incommensurate spin excitations at $(0.5 \pm \delta, 0.5 \pm \delta, 0.5)$ were suggested in $CeCoIn_5$ with δ=0.042(2) at $E = 0.5$ meV [4]. The incommensurate peaks were argued to be a dynamical precursor to the field-induced magnetically ordered Q phase just below H_{c2} [the Q phase orders at $(0.5 \pm \delta, 0.5 \pm \delta, 0.5)$ with δ=0.05(1)] [20]. We were unable to confirm the incommensurate excitations at $E = 0.5$ meV in $CeCoIn_5$ from our experiment at MACS.

To understand how the poorer resolution in our experiments affect our ability to resolve possible incommensurate features, a cut along $[H, H, 0.5]$ at $E = 0.5$ meV is shown in Supplementary Figure 13 and fit with two Guassian peaks separated by 2δ for our data on $CeCoIn_5$. While $\delta = 0.042$ does not provide a good fit to our results, a smaller

splitting of $\delta = 0.033$ can describe our data. The smaller splitting of $\delta = 0.033$ from our data is inconsistent with δ=0.05(1) for the Q phase [20].

Moreover, our inelastic neutron scattering results on $Ce_{1-x}Yb_xCoIn_5$ suggests that the upwards dispersing excitations are ring-like [Fig. 5(c)-5(f)], emanating from $\mathbf{Q}_{\rm AF}$ rather than two peaks at $(0.5 \pm \delta, 0.5 \pm \delta, 0.5)$ as suggested in Ref. [4]. It is possible that there is more than a single contribution to the resonance, with the upward dispersing mode we observe being the dominant feature and an incommensurate mode exists around $E = 0.5$ meV. In this case, the smaller δ we observe comes from bottom of the upward dispersing mode at the commensurate wave vector at $E > 0.5$ meV, which mixes with the incommensurate mode at $E \approx 0.5$ meV due to our poorer resolution. More measurements with better energy and momentum resolutions are needed to clarify the fine details of the resonance around $E = 0.5$ meV. However, our data and analysis are fully consistent with the raw data in Ref. [4], both showing no downward dispersion of the resonance characteristic of a spin-exciton expected for a d-wave superconductor.

Supplementary Note 5: Anisotropy of spin excitations at $\mathbf{Q}_{\rm AF}$

In previous unpolarized and polarized neutron scattering experiments, it was concluded that the resonance spin excitations are exclusively polarized along the c-axis [1, 4]. Here we discuss the possibility of spin excitations polarized along the in-plane longitudinal direction [the [1,1,0] direction at $\mathbf{Q}_{\rm AF} = (0.5, 0.5, 0.5)$] in addition to the c-axis polarized excitations. It should be noted the such anisotropy does not break four fold rotational symmetry of $CeCoIn_5$ as required by the tetragonal crsytal structure, since we are distinguishing between the in-plane longitudinal and transverse directions [the [1,-1,0] direction at $\mathbf{Q}_{\rm AF} = (0.5, 0.5, 0.5)$] [Supplementary Figure 14(b)].

In previous polarized neutron scattering results [4], spin-flip (SF) neutron scattering cross sections $\sigma_x^{\rm SF}$, $\sigma_y^{\rm SF}$, and $\sigma_z^{\rm SF}$ as shown in Supplementary Figure 14(a) were measured where x indicates that neutron polarization direction is parallel to $\mathbf{Q}$, y is perpendicular to $\mathbf{Q}$ but within the scattering plane ($[H, H, L]$ plane), and z is perpendicular to the scattering plane. Since $\sigma_x^{\rm SF}$, $\sigma_y^{\rm SF}$, and $\sigma_z^{\rm SF}$ measures magnetic excitations perpendicular to both $\mathbf{Q}$ and the neutron polarization direction, $\sigma_x^{\rm SF} \propto M_y + M_z$, $\sigma_y^{\rm SF} \propto M_z$, and $\sigma_z^{\rm SF} \propto M_y$ [see Supplementary Figure 14(a)]. At $\mathbf{Q}_{\rm AF} = (0.5, 0.5, 0.5)$, M_y and M_z can be related to magnetic excitations along crystallographic axes by $M_y = M_{110} \sin^2\theta + M_{001}\cos^2\theta$ and $M_z = M_{1\bar{1}0}$, with θ being the angle between $\mathbf{Q}_{\rm AF}$ and $[1, 1, 0]$ [Supplementary Figure 14(a)]. Previous polarized neutron scattering measurements [4] conclusively demonstrated vanishing intensity of M_z compared to M_y, or $M_{1\bar{1}0} \approx 0$. It was then argued that due to tetragonal symmetry of $CeCoIn_5$, $M_{110} = M_{1\bar{1}0}$ and so only M_{001} is present [4]. However, this analysis did not make the distinction between the in-plane longitudinal and transverse directions that is allowed by four fold symmetry of the system, and the polarized experiment only ruled out the excitations polarized along the in-plane transverse direction. The presence of spin-orbit coupling can induce in-plane spin excitation anisotropy, as seen in the tetragonal phase of iron pnictides [21].

From L-dependence obtained in unpolarized neutron scattering measurements, it was also argued that there are only excitations polarized along the c-axis [1]. However M_{110} contributes little to the total intensity near $L = 0.5$, but becomes significant for $L = 1.5$ [Supplementary Figure 14(c)], where neutron absorption becomes extremely strong since either $k_{\rm i}$ or $k_{\rm f}$ will be close to $[1, 1, 0]$ [Supplementary Fig. 15(a) and (b)], which does not happen for $L = 0.5$ [Supplementary Figures 15(c) and (d)]. The scattering triangles are shown for these cases together with the sample (gray slabs) in Supplementary Figure 15. When $L = 0.5$, for both scattering geometries [Supplementary Figures 15(c) and (d)], both directions of $k_{\rm i}$ and $k_{\rm f}$ are far away from $[1, 1, 0]$. When $L = 1.5$, either $k_{\rm i}$ or $k_{\rm f}$ becomes close to $[1, 1, 0]$ resulting in the neutron beam having to transverse more of the sample resulting in much stronger absorption.

This means even if there is a significant M_{110} contribution, it would be very difficult to observe it in either previous [1] or our work. Given the many crystals required for inelastic neutron scattering experiments (Supplementary Fig. 1), it would be rather difficult to estimate accurately the neutron absorption cross section and determine the M_{110} component. In conclusion, while $M_{1\bar{1}0}$ has been eliminated by polarized neutron scattering results [4], the presence of M_{110} along with M_{001} has not been ruled out by current experimental results and its presence is important for understanding the splitting of the resonance mode under an applied magnetic field.

[1] Stock, C., Broholm, C., Hudis, J., Kang, H. J., and Petrovic, C., Spin resonance in the d-Wave superconductor $CeCoIn_5$, Phys. Rev. Lett. **100**, 087001 (2008).

[2] Das, P *et al.*, Magnitude of the magnetic exchange interaction in the heavy-Fermion antiferromagnet $CeRhIn_5$, Phys. Rev. Lett. **113**, 246404 (2014).

[3] Stock, C., Rodriguez-Rivera, J. A., Schmalzl, K., Rodriguez, E. E., Stunault, A., and Petrovic, C., Single to multiquasi-particle excitations in the itinerant helical magnet $CeRhIn_5$, Phys. Rev. Lett. **114**, 247005 (2015).

[4] Raymond, S. and Lapertot, G., Ising Incommensurate Spin Resonance of $CeCoIn_5$: A Dynamical Precursor of the Q Phase, Phys. Rev. Lett. **115**, 037001 (2015).

[5] Eremin, I., Morr, D.K., Chubukov A.V., Bennemann K.H., and Norman, M.R., Novel resonance mode in $d_{x^2-y^2}$-wave supercondutors. Phys. Rev. Lett. **94**, 147001 (2005).

[6] Fong, H.F. *et al.*, Phonon and magnetic neutron scattering at 41 meV in $YBa_2Cu_3O_7$. Phys. Rev. Lett. **75**, 316-319 (1995).

[7] Abanov Ar. and Chubukov A.V., A relation between the resonance neutron peak and ARPES data in cuprates. Phys. Rev. Lett. **83**, 1652-1655 (1999).

[8] Brinckmann, J. and Lee, P.A., Slave boson approach to neutron scattering in $YBa_2Cu_3O_{6+y}$ superconductors. Phys. Rev. Lett. **82**, 2915-2918 (1999).

[9] Kao Y.-J., Si Q., and Levin, K., Frequency evolution of neutron peaks below T_c: Commensurate and incommensurate structure in $La_{0.85}Sr_{0.15}CuO_4$ and $YBa_2Cu_3O_{6.6}$. Phys. Rev. B **61**, R11898(R) (2000).

[10] Onufrieva, F. and Pfeuty, P., Spin dynamics of a two-dimensional metal in a superconducting state: Application to the high-T_c cuprates. Phys. Rev. B **65**, 054515 (2002).

[11] Manske D., Eremin, I., and Bennemann, K.H., Analysis of the resonance peak and magnetic coherence seen in inelastic neutron scattering of cuprate superconductors: A consistent picture with tunneling and conductivity data. Phys. Rev. B **63**, 054517 (2001).

[12] Norman, M.R., Relation of neutron incommensurability to electronic structure in high-temperature superconductors. Phys. Rev. B **61**, 14751 (2000).

[13] Norman, M.R., Magnetic collective mode dispersion in high-temperature superconductors. Phys. Rev. B **63**, 092509 (2001).

[14] Chubukov, A., Janko, B., and Tchernyshov, O., Dispersion of the neutron resonance in cuprate superconductors. Phys. Rev. B **63**, 180507(R) (2001).

[15] Van Dyke, J., Massee, F., Allan, M. P., Davis, J. C., Petrovic, C., and Morr, D. K., Direct evidence for a magnetic f-electron-mediated pairing mechanism of heavy-fermion superconductivity in $CeCoIn_5$, PNAS **111**, 11663-11667 (2014).

[16] Bianchi, A., Movshovich, R., Vekhter, I., Pagliuso, P. G. and Sarrao, J. L. Avoided antiferromagnetic order and quantum critical point in $CeCoIn_5$, Phys. Rev. Lett. **91**, 257001 (2003).

[17] Petrovic, C., Bud'ko, S. L., Kogan, V. G., and Canfield, P. C., Effects of La substitution on the superconducting state of $CeCoIn_5$, Phys. Rev. B **66**, 054534 (2002).

[18] Morr, D.K. and Pines, D., The resonance peak in cuprate supercondutors. Phys. Rev. Lett. **81**, 1086-1089 (1998).

[19] Kim, M. G. *et al.*, Magnonlike dispersion of spin resonance in Ni-doped $BaFe_2As_2$, Phys. Rev. Lett. **110**, 177002 (2013).

[20] Kenzelmann, M. *et al.*, Evidence for a Magnetically Driven Superconductivity Q Phase of $CeCoIn_5$, Phys. Rev. Lett. **104**, 127001 (2010).

[21] Luo , H. et al., Spin Excitation Anisotropy as a Probe of Orbital Ordering in the Paramagnetic Tetragonal Phase of Superconducting $BaFe_{1.904}Ni_{0.096}As_2$, Phys. Rev. Lett. **111**, 107006 (2013).
